# 5,6-Dimethyl-2-(pyridin-2-yl)-1-[(pyri­din-2-yl)meth­yl]-1*H*-benzimidazole

**DOI:** 10.1107/S1600536814003870

**Published:** 2014-02-26

**Authors:** David K. Geiger, Matthew R. DeStefano

**Affiliations:** a Department of Chemistry, State University of New York-College at Geneseo, 1 College Circle, Geneseo, NY 14454, USA

## Abstract

The title compound, C_20_H_18_N_4_, was obtained *via* the condensation of 4,5-di­methyl­benzene-1,2-di­amine with pyridine-2-carbaldehyde. The plane of the 2-(pyridin-2-yl) substitutent is canted by 2.75 (11)° from the plane of the benzimidazole system. The mol­ecule exhibits an *S*(6) C—H⋯N intra­molecular hydrogen-bond motif. In the crystal, C—H⋯N hydrogen bonds link pairs of mol­ecules related by a crystallographic inversion center, forming *R*_2_^2^(20) rings. Additional weak C—H⋯N hydrogen bonds result in *C*(9) chains parallel to [001].

## Related literature   

Reich *et al.* (2004 ▶) provide examples of inter­molecular aldimine coupling. For a discussion of the biological activity of benzimidazole derivatives, see: López-Rodríguez *et al.* (1999 ▶); Horton *et al.* (2003 ▶). For the structure of 2-(pyridin-4-yl)-1*H*-benzimidazole, see: Geiger & Bond (2013 ▶), and for its trihydrate, see: Huang *et al.* (2004 ▶). For the structure of 5,6-di­methyl­benzimidazole, see: Lee & Scheidt (1986 ▶).
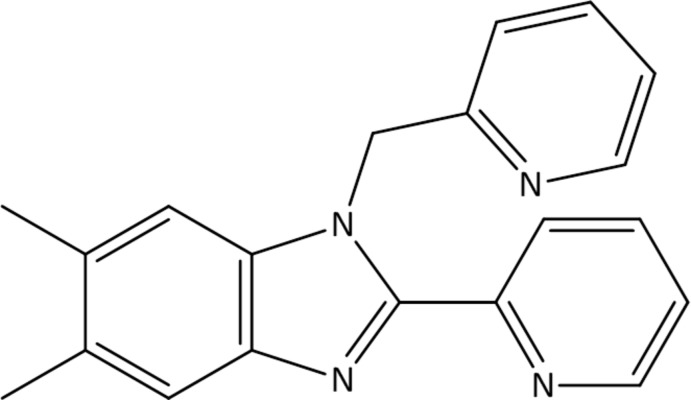


## Experimental   

### 

#### Crystal data  

C_20_H_18_N_4_
*M* *_r_* = 314.38
Monoclinic, 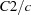

*a* = 35.544 (4) Å
*b* = 6.1194 (5) Å
*c* = 16.5050 (19) Å
β = 113.273 (4)°
*V* = 3297.9 (6) Å^3^
*Z* = 8
Mo *K*α radiation
μ = 0.08 mm^−1^
*T* = 200 K
0.60 × 0.30 × 0.06 mm

#### Data collection  

Bruker SMART X2S benchtop diffractometer
Absorption correction: multi-scan (*SADABS*; Bruker, 2013 ▶) *T*_min_ = 0.45, *T*_max_ = 1.00
8660 measured reflections
3501 independent reflections
2402 reflections with *I* > 2σ(*I*)
*R*_int_ = 0.061

#### Refinement  

*R*[*F* ^2^ > 2σ(*F* ^2^)] = 0.056
*wR*(*F* ^2^) = 0.154
*S* = 1.03
3501 reflections
219 parameters
H-atom parameters constrained
Δρ_max_ = 0.28 e Å^−3^
Δρ_min_ = −0.26 e Å^−3^

### 

Data collection: *APEX2* (Bruker, 2013 ▶); cell refinement: *SAINT* (Bruker, 2013 ▶); data reduction: *SAINT*; program(s) used to solve structure: *SHELXS97* (Sheldrick, 2008 ▶); program(s) used to refine structure: *SHELXL2013* (Sheldrick, 2008 ▶); molecular graphics: *PLATON* (Spek, 2009 ▶); software used to prepare material for publication: *publCIF* (Westrip, 2010 ▶).

## Supplementary Material

Crystal structure: contains datablock(s) global, I. DOI: 10.1107/S1600536814003870/fk2079sup1.cif

Structure factors: contains datablock(s) I. DOI: 10.1107/S1600536814003870/fk2079Isup2.hkl

Supporting information file. DOI: 10.1107/S1600536814003870/fk2079Isup3.mol

Supporting information file. DOI: 10.1107/S1600536814003870/fk2079Isup4.cml

CCDC reference: 987895

Additional supporting information:  crystallographic information; 3D view; checkCIF report

## Figures and Tables

**Table 1:** Hydrogen-bond geometry (Å, °)

*D*—H⋯*A*	*D*—H	H⋯*A*	*D*⋯*A*	*D*—H⋯*A*
C13—H13*A*⋯N3	0.99	2.35	2.948 (3)	118
C11—H11⋯N4^i^	0.95	2.61	3.315 (3)	131
C17—H17⋯N2^ii^	0.95	2.74	3.368 (3)	125

Symmetry codes: (i) 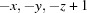; (ii) 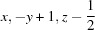.

**Fig. 1.**
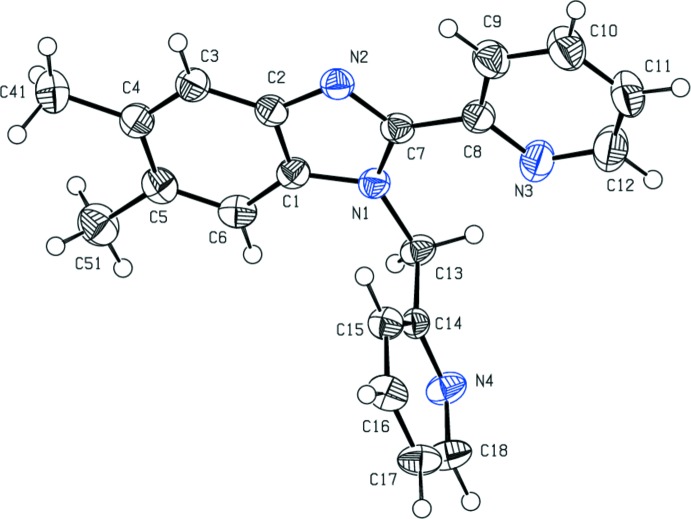
Molecular structure of the title compound. Anisotropic displacement parameters are displayed at the 50% probability level.

**Fig. 2.**
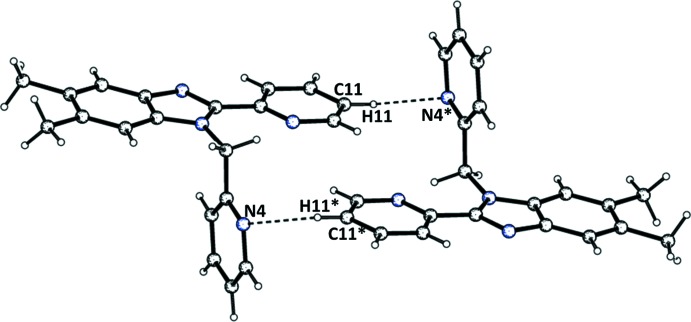
Pairs of molecules related by a crystallographic inversion center. The second molecule* is generated by the operation -x, -y, 1 - z. Dashed lines denote the C11—H11···N4 hydrogen bonds.

**Fig. 3.**
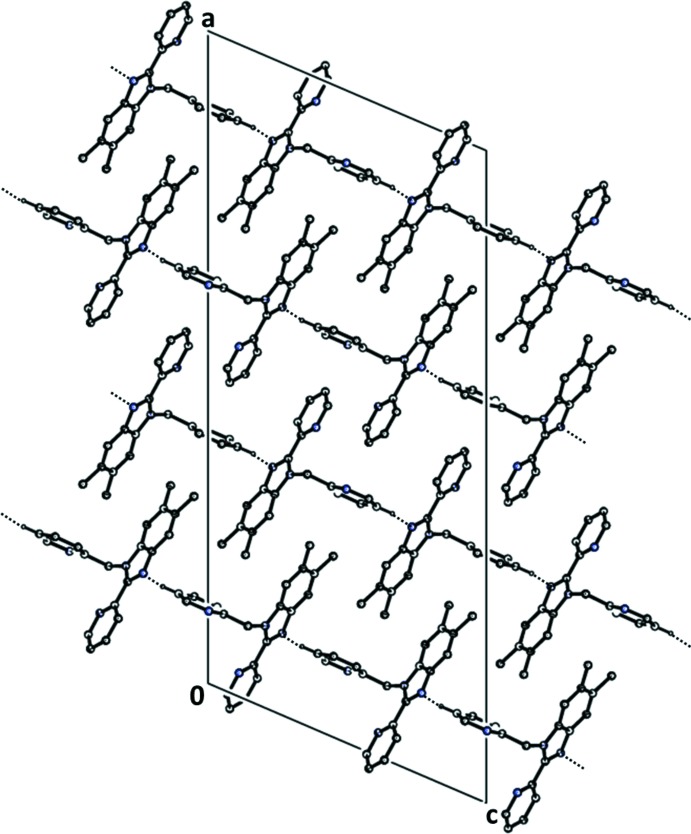
Crystal packing viewed along b-axis with the intermolecular C17—H17···N2 hydrogen bonds (dashed lines) resulting in infinite chains parallel to [001]. Hydrogen atoms, except H17, have been omitted for clarity.

## supplementary crystallographic information

### 1. Comment

Benzimidazole derivatives are of interest because of their pharmacological uses. Examples include inhibitors of serotonin activated neurotransmission drugs (López-Rodríguez *et al.*, 1999) and antiarrhythmic, antihistamine, antiulcer, anticancer, fungicidal, and anthelmintical drugs (Horton *et al.*, 2003). The title compound was prepared as part of our efforts to prepare benzimidazole analogues which have substitutents capable of binding metals (Geiger & Bond, 2013).

The benzimidazole ring system is planar with the largest deviation from planarity for N1 of 0.0173 (13) Å. The largest deviation from planarity in the 2-(pyridin-2-yl) substituent occurs for C9 of 0.0030 (17) Å. The pyridine ring and the benzimidazole ring system are almost coplanar. The angle between the two mean planes is 2.75 (11)°. The 2-(pyridin-2-yl) substituent N atom is *syn* to the 1-(pyridin-2-ylmethyl)substituent, resulting in a weak C—H···N *S*(6) intramolecular hydrogen-bond motif with a C13···N3 distance of 2.948 (3) Å and a C13—H13A···N3 angle of 118.4°. The 1-(pyridin-2-ylmethyl) substituent pyridine ring exhibits a high degree of planarity. The largest deviation is 0.0079 (13) Å for N4.

The solid-state structure displays extensive intermolecular interactions. Pairs of molecules related by crystallographic inversion centers are joined by two weak C11—H11···N4 H-bonds. As shown in Figure 2, the result is an *R*_2_^2^(20) ring with C···N = 3.315 (3) Å and a C—H···N angle of 131°.

Additionally, weak C17—H17···N2 interactions link molecules into *C*(9) chains parallel to [001] (Figure 3). The C—N nonbonded contact is 3.368 (3) Å and the C—H···N angle is 125°.

### 2. Experimental

4,5-dimethyl-1,2-diaminobenzene (2.00 g, 14.7 mmole) was stirred in absolute ethanol (60 ml) for five minutes under nitrogen. 2-pyridinecarboxaldehyde (2.80 ml, 3.15 g, 29.4 mmole) was added dropwise to the reaction mixture with stirring at room temperature. After 24 h, the solution had turned from red to orange with the formation of a precipitate. The reaction mixture was chilled and then filtered using an HPLC grade filter and washed with water. The orange solid was dried yielding 1.37 g (29.7% yield) of pure product. ^1^H NMR (400 MHz, CDCl_3_, p.p.m.): 2.33 (*s*, 3H), 2.38 (*s*, 3H), 6.24 (*s*, 2H), 6.83 (*d*, 1H), 7.11 (*s*, 1H), 7.14 (*t*, 1H), 7.27 (*t*, 1H), 7.47 (*t*, 1H), 7.62 (*s*, 1H), 7.81 (*t*, 1H), 8.43 (*d*, 1H), 8.53 (*d*, 1H), 8.60 (*d*, 1H). ^13^C NMR (CDCl_3_, p.p.m.): 20.4, 20.7, 51.1, 110.7, 120.1, 120.8, 122.2, 123.6, 124.3, 132.0, 133.2, 135.4, 136.8, 136.9, 141.4, 148.6, 149.1, 149.1, 150.6, 157.8.

Single crystals suitable for X-ray diffraction were obtained *via* vapor diffusion of hexane into an ethanol solution of the product at ambient temperature.

### 3. Refinement

All hydrogen atoms were observed in difference fourier maps. The H atoms were refined using a riding model with a C—H distance of 0.99 Å for the methylene carbon atoms, 0.98 Å for the methyl carbon atoms and 0.95 Å for the phenyl and pyridine carbon atoms. The methyl C—H hydrogen atom isotropic displacement parameters were set using the approximation *U*_iso_ = 1.5*U*_eq_. All other C—H hydrogen atom isotropic displacement parameters were set using the approximation *U*_iso_ = 1.2*U*_eq_.

### Crystal data

**Table:**

C_20_H_18_N_4_	*F*(000) = 1328
*M**_r_* = 314.38	*D*_x_ = 1.266 Mg m^−^^3^
Monoclinic, *C*2/*c*	Mo *K*α radiation, λ = 0.71073 Å
*a* = 35.544 (4) Å	Cell parameters from 3800 reflections
*b* = 6.1194 (5) Å	θ = 2.5–26.8°
*c* = 16.5050 (19) Å	µ = 0.08 mm^−^^1^
β = 113.273 (4)°	*T* = 200 K
*V* = 3297.9 (6) Å^3^	Prism, colourless
*Z* = 8	0.60 × 0.30 × 0.06 mm

### Data collection

**Table:**

Bruker SMART X2S benchtop diffractometer	3501 independent reflections
Radiation source: XOS X-beam microfocus source	2402 reflections with *I* > 2σ(*I*)
Doubly curved silicon crystal monochromator	*R*_int_ = 0.061
Detector resolution: 8.3330 pixels mm^-1^	θ_max_ = 27.1°, θ_min_ = 2.5°
ω scans	*h* = −45→45
Absorption correction: multi-scan (*SADABS*; Bruker, 2013)	*k* = −7→6
*T*_min_ = 0.45, *T*_max_ = 1.00	*l* = −21→21
8660 measured reflections	

### Refinement

**Table:**

Refinement on *F*^2^	Primary atom site location: structure-invariant direct methods
Least-squares matrix: full	Secondary atom site location: difference Fourier map
*R*[*F*^2^ > 2σ(*F*^2^)] = 0.056	Hydrogen site location: inferred from neighbouring sites
*wR*(*F*^2^) = 0.154	H-atom parameters constrained
*S* = 1.03	*w* = 1/[σ^2^(*F*_o_^2^) + (0.0585*P*)^2^ + 1.1674*P*] where *P* = (*F*_o_^2^ + 2*F*_c_^2^)/3
3501 reflections	(Δ/σ)_max_ < 0.001
219 parameters	Δρ_max_ = 0.28 e Å^−^^3^
0 restraints	Δρ_min_ = −0.26 e Å^−^^3^

### Special details

**Table:**

Geometry. All e.s.d.'s (except the e.s.d. in the dihedral angle between two l.s. planes) are estimated using the full covariance matrix. The cell e.s.d.'s are taken into account individually in the estimation of e.s.d.'s in distances, angles and torsion angles; correlations between e.s.d.'s in cell parameters are only used when they are defined by crystal symmetry. An approximate (isotropic) treatment of cell e.s.d.'s is used for estimating e.s.d.'s involving l.s. planes.
Refinement. Refinement of *F*^2^ against ALL reflections. The weighted *R*-factor *wR* and goodness of fit *S* are based on *F*^2^, conventional *R*-factors *R* are based on *F*, with *F* set to zero for negative *F*^2^. The threshold expression of *F*^2^ > σ(*F*^2^) is used only for calculating *R*-factors(gt) *etc*. and is not relevant to the choice of reflections for refinement. *R*-factors based on *F*^2^ are statistically about twice as large as those based on *F*, and *R*- factors based on ALL data will be even larger.

### Fractional atomic coordinates and isotropic or equivalent isotropic displacement parameters (Å^2^)

**Table:**

	*x*	*y*	*z*	*U*_iso_*/*U*_eq_	
N1	0.12573 (5)	0.1608 (2)	0.70519 (10)	0.0282 (4)	
N2	0.12580 (5)	0.4886 (3)	0.76699 (11)	0.0310 (4)	
N3	0.03658 (5)	0.2057 (3)	0.61214 (13)	0.0482 (5)	
N4	0.11122 (5)	−0.1414 (3)	0.50291 (12)	0.0366 (4)	
C1	0.16607 (6)	0.2056 (3)	0.76024 (13)	0.0289 (4)	
C2	0.16544 (6)	0.4083 (3)	0.79875 (13)	0.0298 (4)	
C3	0.20184 (6)	0.4972 (3)	0.85981 (14)	0.0345 (5)	
H3	0.2016	0.635	0.8861	0.041*	
C4	0.23817 (6)	0.3845 (3)	0.88192 (14)	0.0342 (5)	
C5	0.23848 (6)	0.1778 (3)	0.84098 (14)	0.0355 (5)	
C6	0.20272 (6)	0.0883 (3)	0.78064 (14)	0.0335 (5)	
H6	0.2029	−0.0487	0.7537	0.04*	
C7	0.10284 (6)	0.3384 (3)	0.71141 (13)	0.0299 (4)	
C8	0.05821 (6)	0.3659 (3)	0.66471 (14)	0.0333 (5)	
C9	0.04023 (7)	0.5580 (4)	0.67726 (17)	0.0437 (6)	
H9	0.0565	0.6686	0.7156	0.052*	
C10	−0.00146 (7)	0.5856 (4)	0.63336 (18)	0.0517 (6)	
H10	−0.0143	0.7159	0.6407	0.062*	
C11	−0.02436 (7)	0.4211 (4)	0.57853 (17)	0.0524 (6)	
H11	−0.0531	0.4356	0.5475	0.063*	
C12	−0.00429 (7)	0.2366 (4)	0.57030 (18)	0.0554 (7)	
H12	−0.0201	0.1234	0.5329	0.067*	
C13	0.11412 (6)	−0.0225 (3)	0.64333 (13)	0.0310 (4)	
H13A	0.0858	−0.0675	0.6326	0.037*	
H13B	0.1324	−0.1478	0.6704	0.037*	
C14	0.11648 (5)	0.0312 (3)	0.55631 (12)	0.0254 (4)	
C15	0.12293 (6)	0.2405 (3)	0.53268 (13)	0.0315 (5)	
H15	0.1266	0.3593	0.5721	0.038*	
C16	0.12391 (6)	0.2736 (3)	0.45063 (15)	0.0371 (5)	
H16	0.1286	0.4156	0.4332	0.045*	
C17	0.11803 (7)	0.0989 (3)	0.39455 (14)	0.0399 (5)	
H17	0.1183	0.1176	0.3377	0.048*	
C18	0.11168 (8)	−0.1032 (4)	0.42288 (15)	0.0451 (6)	
H18	0.1074	−0.2233	0.3838	0.054*	
C41	0.27737 (6)	0.4761 (4)	0.94972 (17)	0.0465 (6)	
H41A	0.2716	0.6146	0.9724	0.07*	
H41B	0.297	0.5013	0.9224	0.07*	
H41C	0.289	0.372	0.9984	0.07*	
C51	0.27837 (7)	0.0560 (4)	0.86530 (17)	0.0480 (6)	
H51A	0.2736	−0.0797	0.8311	0.072*	
H51B	0.2896	0.0213	0.9284	0.072*	
H51C	0.2979	0.1472	0.8522	0.072*	

### Atomic displacement parameters (Å^2^)

**Table:**

	*U*^11^	*U*^22^	*U*^33^	*U*^12^	*U*^13^	*U*^23^
N1	0.0317 (8)	0.0268 (9)	0.0281 (9)	−0.0039 (7)	0.0139 (7)	−0.0029 (7)
N2	0.0330 (8)	0.0303 (9)	0.0319 (10)	−0.0014 (7)	0.0151 (7)	−0.0027 (7)
N3	0.0370 (10)	0.0491 (12)	0.0518 (13)	−0.0056 (9)	0.0103 (9)	−0.0130 (10)
N4	0.0512 (10)	0.0269 (9)	0.0325 (10)	−0.0042 (8)	0.0173 (8)	−0.0045 (8)
C1	0.0338 (10)	0.0298 (11)	0.0237 (10)	−0.0032 (8)	0.0119 (8)	0.0023 (8)
C2	0.0323 (10)	0.0297 (11)	0.0298 (11)	−0.0004 (8)	0.0148 (8)	0.0025 (8)
C3	0.0363 (10)	0.0311 (11)	0.0375 (13)	−0.0045 (9)	0.0161 (9)	−0.0048 (9)
C4	0.0360 (11)	0.0357 (11)	0.0319 (12)	−0.0044 (9)	0.0143 (9)	0.0024 (9)
C5	0.0366 (11)	0.0362 (12)	0.0358 (12)	0.0027 (9)	0.0165 (9)	0.0086 (10)
C6	0.0413 (11)	0.0270 (10)	0.0348 (12)	0.0028 (9)	0.0177 (9)	0.0015 (9)
C7	0.0347 (10)	0.0313 (11)	0.0267 (11)	−0.0021 (8)	0.0155 (8)	0.0003 (9)
C8	0.0333 (10)	0.0379 (12)	0.0298 (11)	−0.0050 (9)	0.0138 (9)	0.0002 (9)
C9	0.0394 (12)	0.0399 (13)	0.0543 (15)	−0.0011 (10)	0.0210 (11)	−0.0060 (11)
C10	0.0393 (12)	0.0505 (14)	0.0666 (18)	0.0025 (11)	0.0224 (12)	−0.0015 (13)
C11	0.0298 (11)	0.0626 (16)	0.0607 (17)	−0.0003 (11)	0.0134 (11)	0.0003 (13)
C12	0.0367 (12)	0.0593 (16)	0.0602 (18)	−0.0044 (11)	0.0082 (11)	−0.0119 (13)
C13	0.0401 (10)	0.0241 (10)	0.0303 (11)	−0.0057 (8)	0.0154 (9)	−0.0039 (8)
C14	0.0222 (8)	0.0240 (10)	0.0284 (10)	0.0005 (7)	0.0084 (7)	−0.0018 (8)
C15	0.0380 (10)	0.0244 (10)	0.0346 (12)	−0.0002 (8)	0.0169 (9)	−0.0002 (8)
C16	0.0465 (12)	0.0291 (11)	0.0415 (13)	0.0054 (9)	0.0236 (10)	0.0082 (9)
C17	0.0543 (13)	0.0425 (13)	0.0284 (12)	0.0073 (10)	0.0222 (10)	0.0047 (10)
C18	0.0663 (15)	0.0369 (12)	0.0358 (13)	0.0026 (11)	0.0241 (11)	−0.0092 (10)
C41	0.0340 (11)	0.0510 (14)	0.0512 (15)	−0.0066 (10)	0.0135 (11)	−0.0021 (11)
C51	0.0426 (12)	0.0496 (14)	0.0510 (16)	0.0097 (11)	0.0174 (11)	0.0069 (12)

### Geometric parameters (Å, º)

**Table:**

N1—C7	1.385 (2)	C10—C11	1.383 (4)
N1—C1	1.388 (2)	C10—H10	0.95
N1—C13	1.462 (2)	C11—C12	1.371 (3)
N2—C7	1.327 (2)	C11—H11	0.95
N2—C2	1.385 (2)	C12—H12	0.95
N3—C8	1.333 (3)	C13—C14	1.508 (3)
N3—C12	1.353 (3)	C13—H13A	0.99
N4—C14	1.341 (2)	C13—H13B	0.99
N4—C18	1.348 (3)	C14—C15	1.384 (3)
C1—C2	1.398 (3)	C15—C16	1.383 (3)
C1—C6	1.407 (3)	C15—H15	0.95
C2—C3	1.398 (3)	C16—C17	1.375 (3)
C3—C4	1.380 (3)	C16—H16	0.95
C3—H3	0.95	C17—C18	1.372 (3)
C4—C5	1.436 (3)	C17—H17	0.95
C4—C41	1.509 (3)	C18—H18	0.95
C5—C6	1.381 (3)	C41—H41A	0.98
C5—C51	1.509 (3)	C41—H41B	0.98
C6—H6	0.95	C41—H41C	0.98
C7—C8	1.474 (3)	C51—H51A	0.98
C8—C9	1.392 (3)	C51—H51B	0.98
C9—C10	1.379 (3)	C51—H51C	0.98
C9—H9	0.95		
			
C7—N1—C1	106.36 (15)	C10—C11—H11	121.0
C7—N1—C13	129.91 (17)	N3—C12—C11	124.1 (2)
C1—N1—C13	122.81 (16)	N3—C12—H12	118.0
C7—N2—C2	105.77 (16)	C11—C12—H12	118.0
C8—N3—C12	117.1 (2)	N1—C13—C14	113.04 (15)
C14—N4—C18	117.07 (17)	N1—C13—H13A	109.0
N1—C1—C2	105.94 (16)	C14—C13—H13A	109.0
N1—C1—C6	132.48 (18)	N1—C13—H13B	109.0
C2—C1—C6	121.58 (18)	C14—C13—H13B	109.0
N2—C2—C1	109.84 (17)	H13A—C13—H13B	107.8
N2—C2—C3	130.41 (18)	N4—C14—C15	122.64 (17)
C1—C2—C3	119.75 (17)	N4—C14—C13	114.13 (16)
C4—C3—C2	119.94 (19)	C15—C14—C13	123.22 (16)
C4—C3—H3	120.0	C16—C15—C14	118.86 (18)
C2—C3—H3	120.0	C16—C15—H15	120.6
C3—C4—C5	119.74 (19)	C14—C15—H15	120.6
C3—C4—C41	120.26 (19)	C17—C16—C15	119.26 (18)
C5—C4—C41	119.99 (18)	C17—C16—H16	120.4
C6—C5—C4	120.85 (18)	C15—C16—H16	120.4
C6—C5—C51	119.74 (19)	C18—C17—C16	118.25 (19)
C4—C5—C51	119.41 (19)	C18—C17—H17	120.9
C5—C6—C1	118.13 (18)	C16—C17—H17	120.9
C5—C6—H6	120.9	N4—C18—C17	123.9 (2)
C1—C6—H6	120.9	N4—C18—H18	118.1
N2—C7—N1	112.07 (17)	C17—C18—H18	118.1
N2—C7—C8	121.56 (17)	C4—C41—H41A	109.5
N1—C7—C8	126.37 (18)	C4—C41—H41B	109.5
N3—C8—C9	122.5 (2)	H41A—C41—H41B	109.5
N3—C8—C7	118.75 (18)	C4—C41—H41C	109.5
C9—C8—C7	118.74 (19)	H41A—C41—H41C	109.5
C10—C9—C8	119.1 (2)	H41B—C41—H41C	109.5
C10—C9—H9	120.4	C5—C51—H51A	109.5
C8—C9—H9	120.4	C5—C51—H51B	109.5
C9—C10—C11	119.2 (2)	H51A—C51—H51B	109.5
C9—C10—H10	120.4	C5—C51—H51C	109.5
C11—C10—H10	120.4	H51A—C51—H51C	109.5
C12—C11—C10	118.0 (2)	H51B—C51—H51C	109.5
C12—C11—H11	121.0		
			
C7—N1—C1—C2	1.14 (19)	C1—N1—C7—C8	180.00 (18)
C13—N1—C1—C2	171.16 (16)	C13—N1—C7—C8	10.9 (3)
C7—N1—C1—C6	−179.63 (19)	C12—N3—C8—C9	0.1 (3)
C13—N1—C1—C6	−9.6 (3)	C12—N3—C8—C7	−179.9 (2)
C7—N2—C2—C1	0.6 (2)	N2—C7—C8—N3	−177.59 (18)
C7—N2—C2—C3	−179.1 (2)	N1—C7—C8—N3	1.5 (3)
N1—C1—C2—N2	−1.1 (2)	N2—C7—C8—C9	2.3 (3)
C6—C1—C2—N2	179.58 (17)	N1—C7—C8—C9	−178.59 (18)
N1—C1—C2—C3	178.63 (18)	N3—C8—C9—C10	−0.5 (3)
C6—C1—C2—C3	−0.7 (3)	C7—C8—C9—C10	179.6 (2)
N2—C2—C3—C4	179.53 (19)	C8—C9—C10—C11	0.5 (4)
C1—C2—C3—C4	−0.1 (3)	C9—C10—C11—C12	−0.1 (4)
C2—C3—C4—C5	1.0 (3)	C8—N3—C12—C11	0.3 (4)
C2—C3—C4—C41	−178.20 (19)	C10—C11—C12—N3	−0.3 (4)
C3—C4—C5—C6	−1.0 (3)	C7—N1—C13—C14	82.2 (2)
C41—C4—C5—C6	178.14 (19)	C1—N1—C13—C14	−85.3 (2)
C3—C4—C5—C51	179.78 (19)	C18—N4—C14—C15	−1.2 (3)
C41—C4—C5—C51	−1.1 (3)	C18—N4—C14—C13	177.96 (18)
C4—C5—C6—C1	0.2 (3)	N1—C13—C14—N4	171.72 (16)
C51—C5—C6—C1	179.41 (18)	N1—C13—C14—C15	−9.1 (3)
N1—C1—C6—C5	−178.49 (19)	N4—C14—C15—C16	0.2 (3)
C2—C1—C6—C5	0.6 (3)	C13—C14—C15—C16	−178.92 (18)
C2—N2—C7—N1	0.2 (2)	C14—C15—C16—C17	0.7 (3)
C2—N2—C7—C8	179.37 (17)	C15—C16—C17—C18	−0.5 (3)
C1—N1—C7—N2	−0.8 (2)	C14—N4—C18—C17	1.4 (3)
C13—N1—C7—N2	−169.90 (17)	C16—C17—C18—N4	−0.6 (4)

### Hydrogen-bond geometry (Å, º)

**Table:**

*D*—H···*A*	*D*—H	H···*A*	*D*···*A*	*D*—H···*A*
C13—H13*A*···N3	0.99	2.35	2.948 (3)	118
C11—H11···N4^i^	0.95	2.61	3.315 (3)	131
C17—H17···N2^ii^	0.95	2.74	3.368 (3)	125

Symmetry codes: (i) −*x*, −*y*, −*z*+1; (ii) *x*, −*y*+1, *z*−1/2.
